# The impact of human immunodeficiency virus infection on cervical preinvasive and invasive neoplasia in South Africa

**DOI:** 10.3332/ecancer.2013.334

**Published:** 2013-07-23

**Authors:** Louis-Jacques van Bogaert

**Affiliations:** National Health Laboratory Service, Polokwane/Mankweng Hospital Complex, University of Limpopo, Polokwane 0700, South Africa

**Keywords:** AIDS-defining illness, cervical neoplasia, HIV, South Africa

## Abstract

**Objectives:**

Sub-Saharan Africa is at the epicentre of the human immunodeficiency virus (HIV) epidemic and has the highest incidence of invasive cervical cancer (ICC) in the world. Access to highly active antiretroviral treatment (HAART) in South Africa is still limited and provided only to nonpregnant women with a CD4+ T-cell count <200 μg/L. We evaluated the relative distribution of cervical preinvasive and invasive neoplasia among HIV-infected (treated or not) and uninfected women in the Limpopo Province of South Africa.

**Methods:**

We compared the consecutive biopsy-diagnosed cervical pathology of 1,023 HIV-infected and 1,023 uninfected women. We investigated the influence of the CD4+ T-cell count and of HAART on the relative distribution of cervical pathology.

**Results:**

There was a significantly higher proportion of cervical intraepithelial neoplasia (CIN)1 (*P *= 0.012) and 2 (*P *= 0.01) but a lower proportion of ICC (*P *= 0.015) among HIV-infected women. Patients on HAART had less CIN1 (*P *= 0.018), 2 (*P *= 0.18) and ICC (*P *= 0.019) that their untreated counterparts. The mean CD4 count was similar regardless of cervical lesions and HAART or no treatment.

**Conclusion:**

Our data support the concept that HIV-infected women exhibit a higher rate of high-grade preinvasive lesions than uninfected controls. However, they have a significantly lower rate of ICC as compared with uninfected counterparts. The inclusion of ICC among acquired immune deficiency syndrome-defining illnesses is questionable.

## Introduction

In 1987, the US Centers for Disease Control and Prevention created a list of clinical conditions called acquired immune deficiency syndrome (AIDS)-defining illnesses (ADI). Most of them were related to microbiological agents, such as viral, fungal, bacterial, and protozoan agents. In 1993, three more were added, such as Kaposi’s sarcoma, lymphoma, and invasive cervical cancer (ICC) [1]. Although the role of human papillomavirus (HPV) in cervical preinvasive and invasive neoplasia was already known, the vast body of investigation currently available was still relatively limited in those days [2]. This may explain why HPV was not listed among the microbiological agents involved in ADI until 2011 [3].

To qualify as an AIDS-defining cancer, the malignancy should be at an increased risk in the HIV-positive population, and the risk should be inversely proportional to the degree of immunosuppression as expressed by the CD4+ T-cell count. In addition, partial immune reversal through highly active antiretroviral treatment (HAART) should decrease the incidence of AIDS-defining cancers [4]. As a whole, the incidence of HPV-induced diseases has increased since the introduction of HAART [5]. Cervical cancer incidence, however, among HIV-infected women in the US has been unchanged since the introduction of HAART [6, 7]. There is no convincing evidence that the HIV epidemic went parallel with an increase in ICC in sub-Saharan Africa [8].

In general, the rate of HPV-associated cancers is higher among people with AIDS; however, the relative risk of ICC linked to immunodepression is only 1.32 [95% confidence interval (CI) 0.96–1.80 and *P *= 0.077] [9, 10]. It has also been speculated that the increased longevity of patients on HAART would result in an increase in ICC among HIV-infected women. Perhaps it is still difficult to witness this within a timespan of ten years. However, the fact that the average age at diagnosis of ICC in HIV-infected women is at least ten years younger than that of HIV-naive women does not support this hypothesis [11].

It is well documented that HIV-infected women are at a risk of carrying one or more high-risk (HR) HPV and tend to have a low clearance rate [12]. Therefore, they are at HR of developing high-grade squamous intraepithelial lesions (HGSIL) or HR cervical intraepithelial neoplasia (CIN) 2+. Whether this places them at increased risk of ICC is controversial [4]. 

## Methods

### Study settings and participants

The study was approved by the Research Ethics Committee of the Polokwane/Mankweng Hospital Complex and the Institutional Review Board of the University of Limpopo at Polokwane. The province has a population in excess of 5,000,000, which is mainly rural. The laboratory receives all surgical pathology specimens (in excess of 10,000 cases/annum) from the public health facilities serving at least 80% of the population. During the five-year study period (2008–2012), 5,500 specimens were cervical in origin and 1,023 (19.0%) were from HIV-infected women.

A consecutive series of cervical biopsies of 1,023 HIV-infected women was prospectively collected over a period of five years (2008–2012). Consecutive cervical biopsies from 1,023 HIV-uninfected women served as controls. The majority of specimens consisted of colposcopydirected large-loop excision of the transformation zone represented by an abnormal cytology, low grade (LG) or HGSIL. Punch biopsies were from symptomatic (contact bleeding and bloody vaginal discharge) women. Information was collected from the laboratory request form and included age, cytological diagnosis, HIV serostatus, CD4+ T-cell count at the time of cervical pathology diagnosis (when available), and HAART (when applicable).

## Laboratory tests

The surgical pathology specimens were routinely processed (10% buffered formalin fixation and paraffin embedding). Four micron sections were stained with haematoxylin and eosin. Preinvasive lesions were subdivided into CIN1, 2 and 3.

## Statistical analysis

Data entries were carried out on site. Statistical analysis was done using GraphPad (Prism, San Diego, CA). We used column statistics, Student’s *t *test, chi-square (*Χ*^2^) analysis for contingency tables, and 95% CIs of proportions. The level of statistical significance was set at *P* < 0.05.

## Results

Table 1 lists the relative distribution of preinvasive and invasive lesions according to HIV serostatus. HIV-positive women had significantly more CIN1 and 2 than HIV-uninfected women (*P *= 0.012 and 0.01). The proportion of CIN3 was similar (*P *= 0.07). HIV-negative patients had significantly more invasive cancers than the HIV positives (*P* = 0.015).

Table 2 lists the relative distribution of cervical neoplasia in HIV-infected women according to the treatment status. Untreated women had fewer preinvasive lesions but a higher rate of ICC than treated women (*P* = 0.019).

Table 3 lists the mean CD4+ T-cell counts of uninfected and untreated infected women. No data were available for HIV-negative CIN1 and 3 cases. Among uninfected women, CD4 counts with CIN2 were significantly higher than with ICC (*t *= 2.2 and *P *= 0.032). Among infected untreated women, there was no difference in CD4 counts between CIN2 and ICC (*t* = 0.93 and *P* = 0.35).

Tables 4 and 5 list the average CD4 counts of HIV-infected women according to the cervical pathology and treatment. No significant difference was found between CD4 counts on HAART or not between CIN1–CIN3 and invasive cancer. CD4 counts were similar with increasing severity regardless of treatment or no treatment. Table 6 shows that the cervical pathology and treatment did not affect the proportion of cases with CD4 counts below 200/μL.

## Discussion

Current South African cervical cancer statistics are lacking, since the latest National Cancer Registry report dates back to 1999 when ICC ranked first among female cancers [13]. The total prevalence of 19.0% of HIV seropositivity in the present series of preinvasive and invasive lesions was similar to the reported incidence of 20.0% HIV infection in the South African general female population between the ages of 15 and 45 [14].

The prevalence of HIV infection in women with ICC varies from 2.7 in Nigeria to 15%, 18% and 21% in Uganda, Kenya and Tanzania, respectively, [8, 15–17]. South African surveys of ICC with known HIV status found that 6.0% and 13.1%, respectively, were HIV positive, showing no excess risk of ICC [18, 19].

According to Bower *et al*, to qualify as ADI, the risk of an AIDS-related malignancy should increase [4]. The odds ratio of the association between HIV and ICC has been estimated to vary from 1.17 to 1.27, 1.32 and 1.60, showing only a small increased risk [6, 8, 10, 18]. Hence, the first requirement is not met as far as ICC is concerned.

Second, the risk of ADI should be inversely proportional to the degree of immunosuppression as expressed by the CD4+ T-cell count [4]. This, however, is a controversial and hotly debated issue. It is a fact that HIV infection results in loss of CD4+ T-cells, followed by an increase of certain but not all HPV-associated anogenital malignancies; cervical cancer, however, is an exception [5, 20]. The mean CD4+ T-cell count in ICC has been shown to be 312/μL, showing that there is less immunosuppression in ICC than other cancers [21]. The fact that HPV is more likely to persist in HIV-infected persons is independent of their CD4 count [7]. It appears to result from an impaired anti-HIV IgA local response [22, 23]. Data suggest that immunosuppression may lead to higher levels of HPV replication with resulting higher HPV DNA levels and increased incidences of LGSIL and HGSIL [24]. However, there have been no similar findings in ICC; cellular genetic changes rather than immunosuppression may be the driving force of progression into ICC [24].

Immunosuppression is most strongly associated with preinvasive lesions; the progression to ICC is not solely associated with immunosuppression [5]. In HIV-infected women, the hazard ratio of developing any preinvasive lesion was 1.2 if the CD4+ T-cell count was >500/μL, which is similar to HIV-uninfected controls [25]. The relative incidence of clearance has been said to decrease with a CD4 count <200 [26]. A Zimbabwean study, however, concluded that only HR HPV, and not HIV, was associated with CIN [27]. The rate of ICC in women with AIDS might be unrelated to immunosuppression and, therefore, not biologically associated with HIV [20].

Third, partial immunity reversal through HAART should decrease the incidence of AIDS-defining malignancies [4]. It has been expected that reversal of immunosuppression following HAART would have a dramatic effect on the natural history of cervical preinvasive lesions [26], but data on the effects of HAART in this regard are mixed [28, 29].

The increased risk of HIV-infected women to CIN2 may be due to different factors: a) high exposure to risk factors (coitarche, number of sex partners, and contraception), b) a direct effect of HIV, or c) a molecular interaction between HIV and HPV [30, 31].

It has been hypothesised that since more women with HIV survive due to access and use of HAART, the incidence of cervical cancer is likely to be increased by the HIV epidemic [32, 33]. However, so far the incidence of cervical cancer among HIV-infected women in the industrialised world has been unchanged since the introduction of HAART [7, 34]. The incidence of HPV-induced diseases has increased rather than decreased since the introduction of HAART; HAART restores the immune response to AIDS-defining opportunistic infections such as Cytomegalovirus and Kaposi’s sarcoma associated virus; 25% of the HIV-infected CIN1 subjects on HAART still progress to CIN2+, and HIV-infected patients with CIN2+ often do not show regression when treated with HAART. Regression of CIN1 and CIN2+ depends on the type of HPV (oncogenic or not) rather than on immunocompetence [5]. The longer survival of HIV-infected patients related to HAART may have a proportionally greater impact on the risk of HPV-related cancers than the partial reversal of immunosuppression that occurs with HAART [20].

The study of HPV-induced cervical neoplasia in HIV-infected women is complex and is riddled with contradictory reports. For instance, it has been reported, as already mentioned, that the incidence of cervical cancer has not decreased in the HAART era and that clinical research has not shown a clear benefit of HAART in decreasing HPV-related cervical disease in HIV-infected women [29, 35]. Other reports, however, claim the contrary [36, 37]. Still others claim the evolution to depend on the degree of immunosuppression [25]. However, contrary to Hawes *et al *[38], no impact in the development of SIL was evidenced for CD4+ T-cell counts by Heard *et al *[39].

The strength of our investigation was that we carried out a large case-control study. The weakness was that we had to rely on the information provided by the request for biopsy diagnosis. Like others, we found a high prevalence of HGSIL. We found no effect of HAART on the relative distribution of preinvasive and invasive neoplasia and no effect of HAART on the degree of immunosuppression.

## Conclusion

Many HIV-associated cancers, such as Kaposi’s sarcoma, lymphomas and HPV-associated head and neck, and lower anogenital squamous cancers, are known or suspected to be ADIs [40]. The numerous data from the literature show that this is not the case with cervical cancer. Our investigation shows that the prevalence of ICC is significantly lower in HIV-infected women than in uninfected women. HAART neither affects the distribution of preinvasive lesions nor the CD4+ cell counts. This suggests that ICC should not be listed among ADIs.

## Conflict of interest

The author declares that he has no conflict of interest.

## Figures and Tables

**Table 1.** Relative distribution of preinvasive and invasive lesions: HIV-negative versus HIV-positive women

Pathology	HIV negative	HIV positive	*P*
CIN1	121 (11.8)^a^	220 (21.5)	0.012
CIN2	77 (7.5)	211 (20.6)	0.010
CIN3	241 (23.6)	279 (27.3)	0.07
Invasive cancer	584 (57.1)	313 (30.6)	0.015
Total	1,023	1,023	

a Values are as numbers (%).

HIV: human immunodeficiency virus.

**Table 2.** Relative distribution of preinvasive and invasive lesions in HIV-positive women: not on HAART versus on HAART

Pathology	HIV positive no HAART	HIV positive on HAART	*P*
CIN1	106 (18.1)^a^	114 (26.1)	0.018
CIN2	102 (17.4)	109 (24.9)	0.018
CIN3	164 (28.0)	115 (26.3)	0.084
Invasive cancer	214 (36.5)	99 (22.7)	0.019
Total	586	437	

a Values are as numbers (%).

HIV: human immunodeficiency virus.

HAART: highly active antiretroviral treatment.

**Table 3.** CD4+ T-cell counts/μL: HIV-negative versus HIV-positive untreated women

Pathology	HIV negative	HIV positive not on HAART	*t*	*P*
CIN1	–	–	–	–
CIN2	1130.5 (426.3) [821.0]^a^ *N* = 12	304.6 (199.0) [258.0] *N* = 32	117.2	<0.0001
CIN3	–	–	–	–
Invasive cancer	849.4 (418.0) [821.0] *N* = 86	356.8 (298.1) [280.0] *N* = 121	9.9	<0.0001

a Values are as mean (SD) [median].

HIV: human immunodeficiency virus.

HAART: highly active antiretroviral treatment.

SD: standard deviation.

N: number of cases.

**Table 4.** CD4+ T-cell counts/μL in HIV-positive women: not on HAART versus HAART

Pathology	Not on HAART	On HAART	*t*	*P*
CIN1	310.2 (213.3) [260.0]^a^ *N* = 30	342.7 (241.2) [275.5] *N* = 6	0.57	0.57
CIN2	304.6 (199.0) [258.0] *N* = 32	284.5 (224.3) [204.0] *N* = 8	0.25	0.80
CIN3	344.8 (274.6) [295.0] *N* = 70	316.2 (260.3) [247.0] *N* = 22	0.28	0.78
Invasive cancer	356.8 (298.1) [280.0] *N* = 121	211.2 (35.2) [225.0] *N* = 6	1.2	0.24

a Values are as mean (SD) [median].

HIV: human immunodeficiency virus.

HAART: highly active antiretroviral treatment.

SD: standard deviation.

N: number of cases.

**Table 5.** Statistical significance of the difference in mean CD4+ T-cell count/μL of preinvasive and invasive lesions on HAART and not on HAART

	Not on HAART		On HAART	
	*t*	*P*	*t*	*P*
CIN1 versus CIN2	0.10	0.92	0.50	0.65
CIN2 versus CIN3	0.62	0.54	0.36	0.76
CIN3 versus invasive cancer	0.17	0.86	0.97	0.34

HAART: highly active antiretroviral treatment.

**Table 6.** Number of cases with CD4+ T-cell count <200/μL

Pathology	Not on HAART	On HAART	*X^2^*	*P*
CIN1	10	2	0.2	0.65
CIN2	9	8	0.7	0.10
CIN3	23	10	1.8	0.18
Invasive cancer	41	2	0.7	0.39

HAART: highly active antiretroviral treatment.
